# Inhibitory effect of miR-377 on the proliferative and invasive behaviors of prostate cancer cells through the modulation of MYC mRNA via its interaction with BCL-2/Bax, PTEN, and CDK4

**DOI:** 10.18632/genesandcancer.236

**Published:** 2024-05-16

**Authors:** Yasamin Azimi, Sara Hajibabaei, Ghazal Azimi, Fatemeh Rahimi-Jamnani, Masoumeh Azizi

**Affiliations:** ^1^Department of Molecular Medicine, Biotechnology Research Center, Pasteur Institute of Iran, Tehran, Iran; ^2^Department of Nanotechnology, Tehran Medical Branch, Islamic Azad University, Tehran, Iran; ^3^Department of Mycobacteriology and Pulmonary Research, Pasteur Institute of Iran, Tehran, Iran

**Keywords:** miR-377, prostate cancer, MYC, PTEN, CDK4

## Abstract

The MYC gene is a regulatory and proto-oncogenic gene that is overexpressed in the majority of prostate cancers (PCa). Numerous studies have indicated that aberrant expression of microRNAs is involved in the initiation and progression of prostate cancer.

In this investigation, we assessed the impact of miR-377 on MYC through luciferase assay. Real-time PCR was employed to determine whether miR-377 could reduce the levels of MYC mRNA in transfected PCa cell lines (PC-3 and DU145) and change in the mRNA levels of BCL-2/Bax, PTEN, and CDK4 as a consequence of MYC downregulation. Moreover, we analyzed the effects of miR-377 on apoptosis, proliferation, cell cycle, and wound healing. Our findings demonstrate that miR-377 effectively targets MYC mRNA, as confirmed by luciferase assay and Real-time PCR. We observed a significant reduction in BCL-2 and CDK4 expression, along with an increase in Bax and PTEN, in prostate cancer cell lines upon MYC suppression. Additionally, elevated levels of miR-377 in PCa cell lines induced apoptosis, inhibited proliferation and migration, and arrested the cell cycle.

Taken together, these results unveil the inhibitory role of miR-377 in MYC function within PCa, thereby suggesting its potential as a therapeutic target for the treatment of this malignancy.

## INTRODUCTION

The frequency of prostate cancer (PCa), which is the second most common malignancy and the third most prevalent cause of cancer fatality in males, is experiencing a rapid increase [1, 2]. The primary causes of mortality remain to be the spread and recurrence of the disease, as well as the emergence of hormone-resistant conditions. As a result, it is of utmost importance to conduct research on the molecular pathways underlying the progression of PCa, as this may potentially lead to the development of a novel approach for targeted treatment of the disease [3].

Non-coding RNAs (ncRNAs) known as micro-RNAs (miRNAs) bind to the 3′untranslated regions of target genes in order to regulate gene expression post-transcriptionally [4]. Numerous studies have indicated that miRNAs play a role in the proliferation of tumors and could potentially be used for the early detection of human malignancies. [5, 6]. For example, data derived from a study demonstrated that the blood serum of patients with prostate cancer, as well as the tumor tissue of mouse prostate, manifest heightened quantities of miR-19b. This suggests that miR-19b might hold significant potential in terms of both diagnostic and predictive applications [7]. The significance of miR-377 has been sparked due to its ability to repress the growth of diverse cancers [8, 9]. Furthermore, NSCLC samples have demonstrated a decrease in expression of miR-377, and its subsequent re-expression inhibits tumor growth by negatively modulating genes involved in the ErbB signaling pathway [10]. Similarly, pancreatic cancer samples have exhibited a decrease in expression of miR-377, and its introduction via transfection can suppress cell growth and enhance apoptosis [11]. Cervical cancer cell research has revealed that an over-expression of miR-377 hampers cell growth and migration, ultimately leading to apoptosis in this type of cancer by targeting FGFR1 [12]. A study conducted on cervical carcinoma unveiled that the expression of miR-377-3p was reduced, suggesting a potential association with unfavorable prognosis in patients with cervical carcinoma. Additionally, in this particular cancer, overexpression of miR-377-3p hindered the invasion and migration of cells. In the context of cervical carcinoma cells, the upregulation of miR-377-3p likewise impeded the epithelial-mesenchymal transition (EMT) process by specifically targeting SGK3. Furthermore, an investigation utilizing prostate cancer cells demonstrated that miR-377 and other miRNAs within the 14q32.31 region are underexpressed in human prostate cancer [13]. Elevated levels of SNHG4 were found to be correlated with lymph node metastases, tumor stage, and poorer overall survival in prostate cancer patients. The regulation of miR-377 expression is governed by the reduction of SNHG4, which also influences the proliferation, migration, and invasion of malignant cells [14].

The oncogene MYC, located on chromosome 8q24, encodes the indispensable transcription factor c-myc, which plays a crucial role in regulating cellular metabolism, proliferation, and programmed cell death. Numerous investigations have demonstrated that MYC is overexpressed in various types of cancer, including prostate cancer, and is closely associated with tumor advancement [15, 16]. In the majority of cases of advanced and metastatic castrate-resistant prostate cancer (mCRPC), there is a notable increase in MYC protein abundance [17]. Furthermore, it has been observed that the MYC gene interacts with other genes, such as BCL-2/Bax, PTEN, and CDK4, which are involved in crucial cellular processes like apoptosis, proliferation, and the cell cycle. In this particular study, we have made the discovery that miR-377 exerts substantial inhibitory effects on proliferation, the cell cycle, and migration, while also inducing apoptosis in prostate cancer cell lines by specifically targeting the oncogene MYC. Our research findings suggest that miR-377 could potentially serve as a valuable therapeutic strategy for the treatment of prostate cancer (PCa).

## RESULTS

### miR-377 can target the 3'-UTR of MYC in prostate cancer cell lines

**Figure 1:**
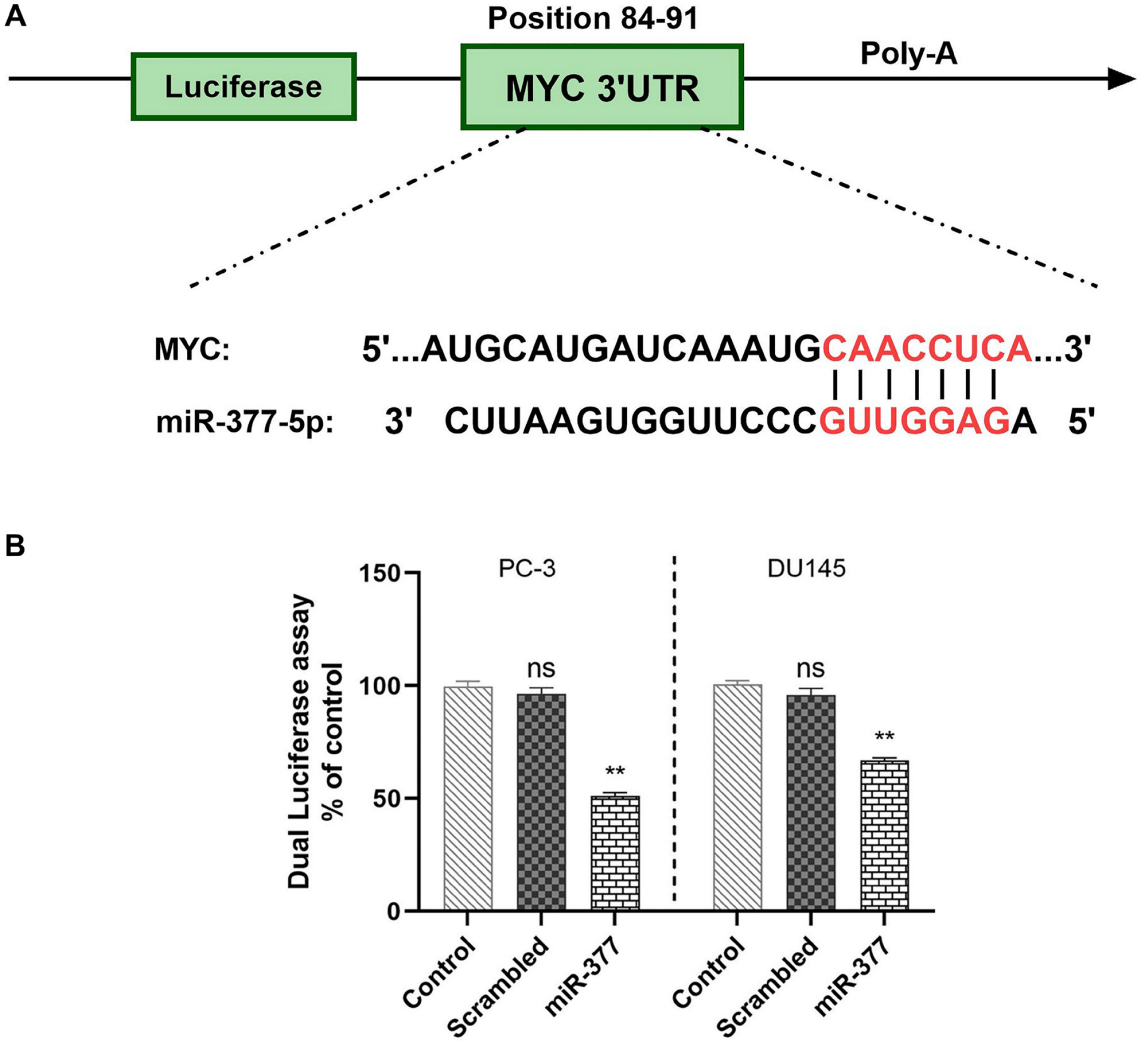
MYC is a direct target of has-miR-77. (**A**) miR-377 has binding sites within the 3′-UTR of the human MYC gene. (**B**) PC-3 and DU145 cells were transfected with Renilla and firefly luciferase expression constructs psiCHECKTM-2 harboring MYC 3′-UTR and miR-377 or scramble-miR. Dual-Luciferase assays were done after 24 hours. The relative Renilla luciferase activity decreased, but the scramble-miR had no effect, ^*^P ≤ 0.05, Mean ± SD.

Using miRDIP (https://ophid.utoronto.ca/mirDIP), miRwalk (http://mirwalk.umm.uni-heidelberg.de/), and TargetScan4.0 (http://www.targetscan.org/), we looked for miR-377 binding sites in the 3′-UTR of MYC. miR-377 was linked to the MYC gene’s 3′-UTR (Figure 1A). To confirm these miR-target contacts, we cloned the MYC complementary site into psiCHECK-2TM to demonstrate that miR-377 targets MYC. PC-3 and DU145 cells were co-transfected by psiCHECKTM-2 vectors containing MYC 3′-UTR or scrambled sequence, and 100 nM of each pre- miR precursor (miR-377 or scrambled miRNAs). miR-377 reduces luciferase activity compared to scramble, as indicated in (Figure 1B). Transfecting miR-377 into PC-3 and DU145 cell lines decreased luciferase activity to 48.4% ± 1.555 and 33.85% ± 1.099 (*P* < 0.05), respectively.

### MYC mRNA downregulated by transfecting miR-377 in prostate cancer cell lines

**Figure 2:**
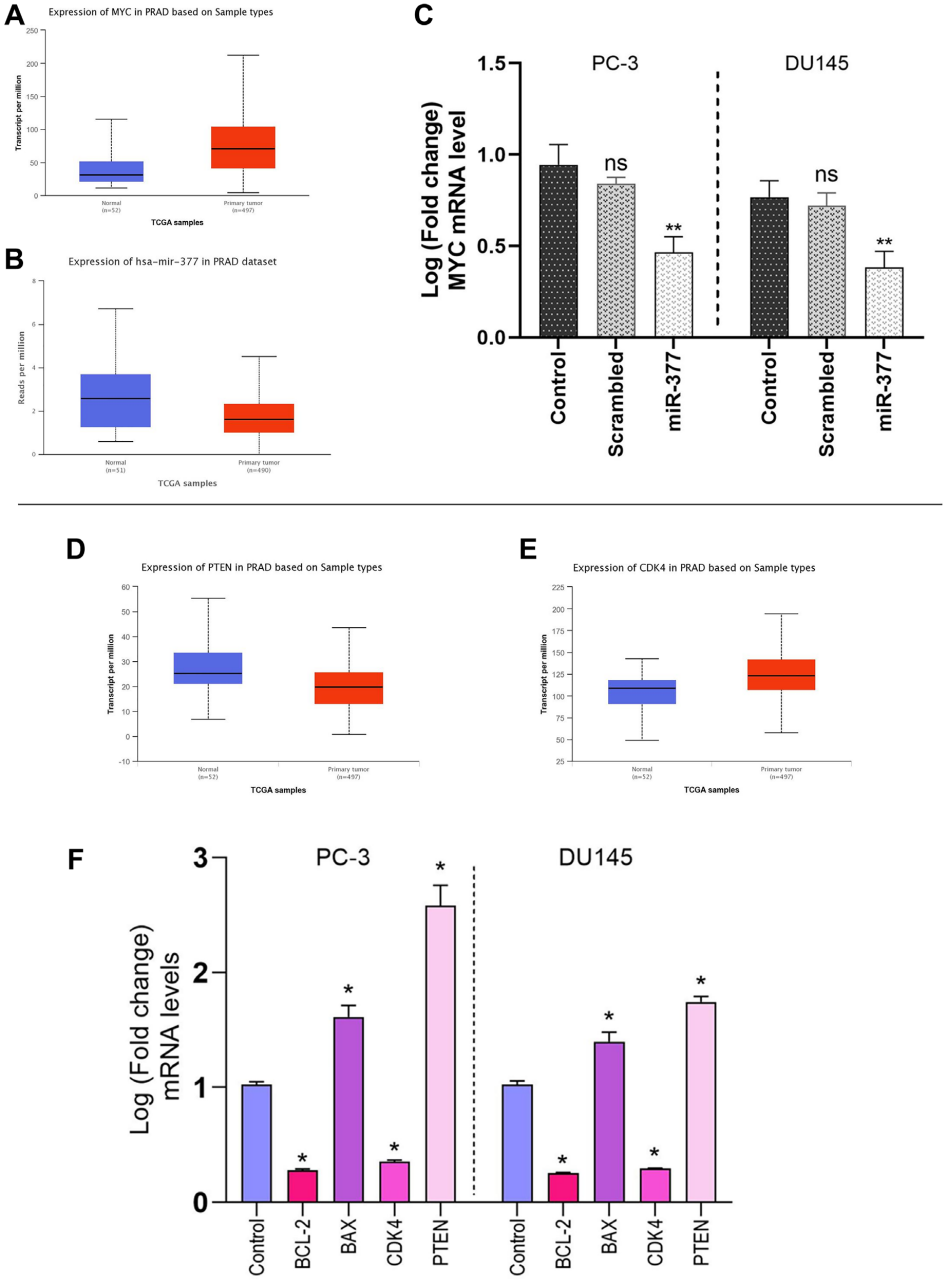
The level of MYC mRNAs following miR-377 or scrambled-miR transfection. (**A**) Analysis of MYC expression in PCa via TGCA samples. (**B**) Analysis of miR-377 expression in PCa via TGCA samples. (**C**) PC-3 and DU145 were transfected with miR-377 or scrambled. After 48 h, MYC expression was evaluated by real-time quantitative PCR. The decrease in relative mRNA expression was evident with miR-377, while no effect was detected with the scrambled-miR. (**D**) Analysis of PTEN expression in PCa via TGCA samples. (**E**) Analysis of CDK4 expression in PCa via TGCA samples. (**F**) BCL-2/Bax, PTEN, and CDK4 mRNA levels in control and miR-377 transfected in PC-3 and DU145 cell lines, ^*^P ≤ 0.05, Mean ± SD.

The results of the differential expression analysis of MYC such as TCGA samples showed that its expression in tumor tissues increased significantly in patients. Also, expression analysis of miR-377 in prostate cancer tissues compared to normal tissues has shown down expression (Figure 2A, 2B). We transfected pre-miR-377 or a scrambled oligonucleotide into PC-3 and DU145 cells to verify the hypothesis that overexpression of miR-377 downregulates MYC mRNA expression in PCa cell lines, and we then evaluated levels of MYC mRNA by quantitative real-time PCR. As shown in (Figure 2C), the overexpression of miR-377 caused reductions in the level of MYC mRNA in PC-3 to 0.4787 ± 0.0802, and in DU145 to 0.3827 ± 0.0720.

### Transfection of miR-377 into PCa cell lines inhibited cell migration due to MYC down-expression

**Figure 3:**
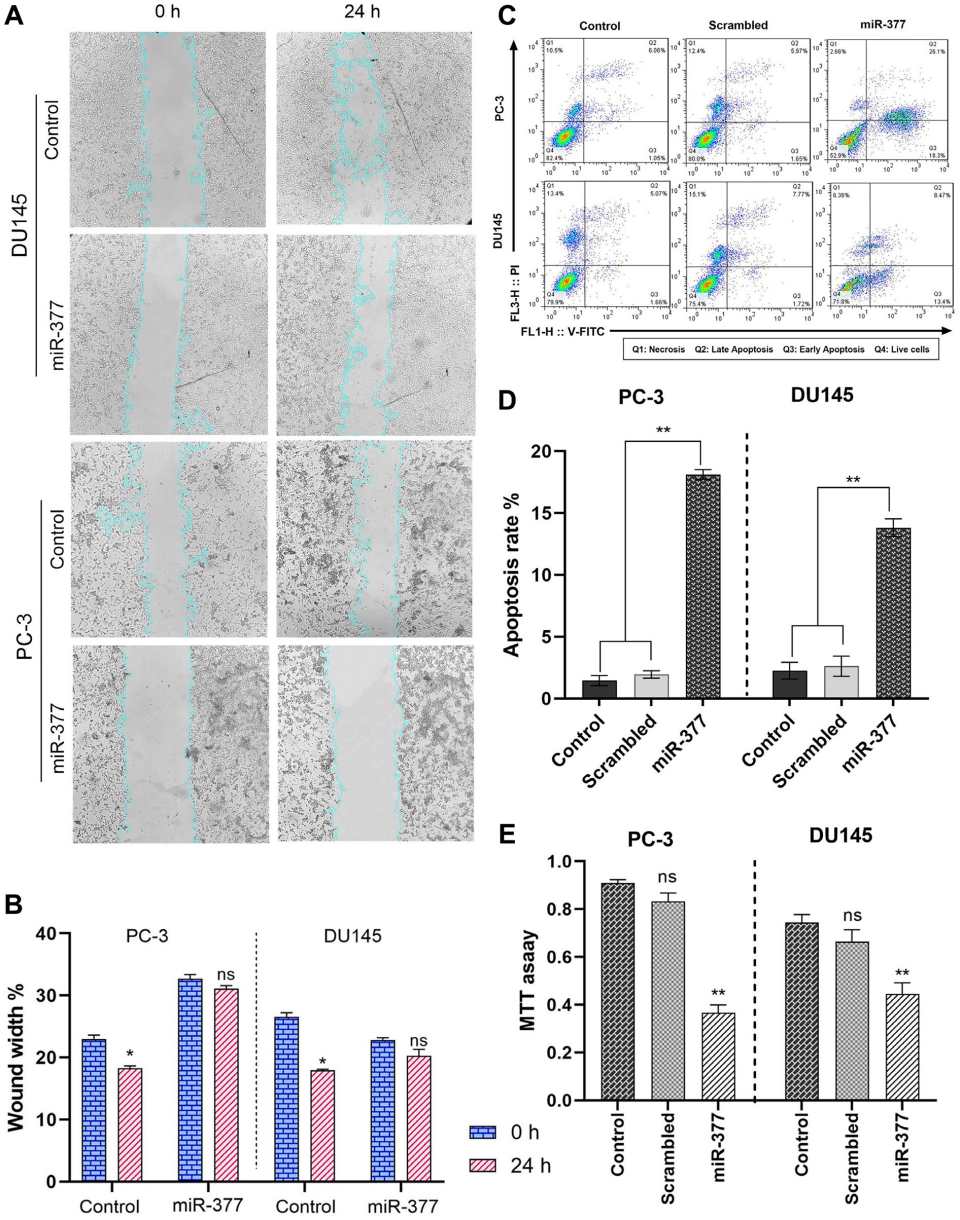
The effects of the miR-377 on cell migration, apoptosis, and MTT of prostate cancer cell lines. (**A**) Compared to control cells, representative images from wound healing assay of PC-3 and DU145 cell cultures 24 h after miR-377 transfection. (**B**) Wound healing assay graph of PC-3 and DU145 cell cultures 24 h after miR-377 transfection demonstrated that cell invasion into the cell-free region (outlined) is abatement compared to control cells. (**C**) Apoptosis induction was investigated using flow cytometry. (**D**) Apoptosis rates are shown in the graphs; Overexpression of miR-377 can induce apoptosis in PC-3 and DU145 cell lines. (**E**) The proliferation of PC-3 and DU145 cells transfected with miR-377, scrambled-miR oligonucleotide and control cells were determined using the MTT assay. Experiments were performed three times, and data are shown as mean ± SD. The results showed that miR-377 could markedly inhibit cancer cell proliferation of PCa cell lines, ^*^P ≤ 0.05, Mean ± SD.

The wound healing experiment is depicted in (Figure 3A, 3B). 24 hours after the cell monolayers were injured, 32.5% of PC-3 cells and 22.4% of DU145 cells had filled the cleared regions without transfection. The adherent cells were made to move into the wound region more hardly after miR-377 transfection. The optimum inhibitory effects of miR-377 on PC-3 and DU145 reached 31.7% and 17.2%, respectively. Cell migration was significantly reduced in both prostate cell lines after transfection.

### Overexpression of miR-377 induced apoptosis in prostate cancer cell lines migration due to MYC down-expression

The impact of miR-377 on apoptosis in PC-3 and DU145 cells was assessed using annexin V and PI in flow cytometry. Compared to controls, the findings showed a rise in the number of cells going through early apoptosis. As shown in (Figure 3C, 3D), control cells for this experiment included un-transfected cells and cells transfected with scrambled oligonucleotides. In cells transfected with miR-377 compared to controls, the apoptosis ratio increased considerably. The percentage of early apoptotic cells increased from 1.05% to 18.3% in PC-3 and from 1.66% to 13.4% in DU145 transfected with miR-377.

### Increased apoptosis is caused by BCL-2 down-expression and Bax over-expression

Real-time PCR analysis revealed that, compared to control cells, miR-377-transfected prostate cancer cell lines had higher levels of Bax expression. Instead, transfected cells had lower levels of BCL-2 expression than the control cells, an anti-apoptotic protein. The results of expression analysis of MYC mRNA down-regulation showed a significant decrease in BCL-2 mRNA and an increase in Bax mRNA levels. (BCL-2 mRNA level shown 0.256 ± 0.0113 and 0.27 ± 0.0253 in PC-3 and DU145 cells, respectively and Bax mRNA level increased 1.61 ± 0.025 in PC-3 and 1.49 ± 0.025 in DU145) (Figure 2F). We discovered that miR-377 dramatically decreased MYC expression after transfection. MYC inhibition reduced the expression of BCL-2 mRNA in PC-3 and DU145 prostate cancer cells. However, miR-377 treatment increased the Bax protein expression in these cells. This demonstrated that the decrease in MYC mRNA levels affected the Bax to BCL-2 ratio in favor of apoptosis.

### Overexpression of miR-377 inhibited cell proliferation in prostate cancer cell lines due to MYC down-expression

An MTT test was used to comprehend miR-377’s function in the proliferation of prostate cancer cell lines. In 96-well plates of PC-3 and DU145 cells, miR-377 or scrambled oligonucleotides were transfected. The proliferation of these cells was assessed 48 hours after transfection in contrast to non-transfected and scrambled cells. In PC-3 and DU145 cell lines, we showed that the miR-377 might considerably (*P* < 0.05) slow down cell proliferation compared to the scrambled and control groups (Figure 3E). Three experiments were conducted.

### Inhibited cell proliferation is caused by PTEN over-expression

The TGCA samples showed that cancer tissues have low PTEN mRNA levels compared to normal tissues (Figure 2D). The analysis of the real-time PCR data showed that the effect of miR-377 on prostate cancer cell lines resulted in a decrease in MYC expression in these cell lines when compared to control cells and an increase in PTEN expression in PC-3 and DU145 (2.54 ± 0.2058 and 1.65 ± 0.06 respectively), due to the inverse relationship between MYC and PTEN expression. Because of this, PTEN’s contribution to cell survival and proliferation has diminished.

### Overexpression of miR-377 inhibited the cell cycle of PCa Cell lines due to MYC down-expression

**Figure 4:**
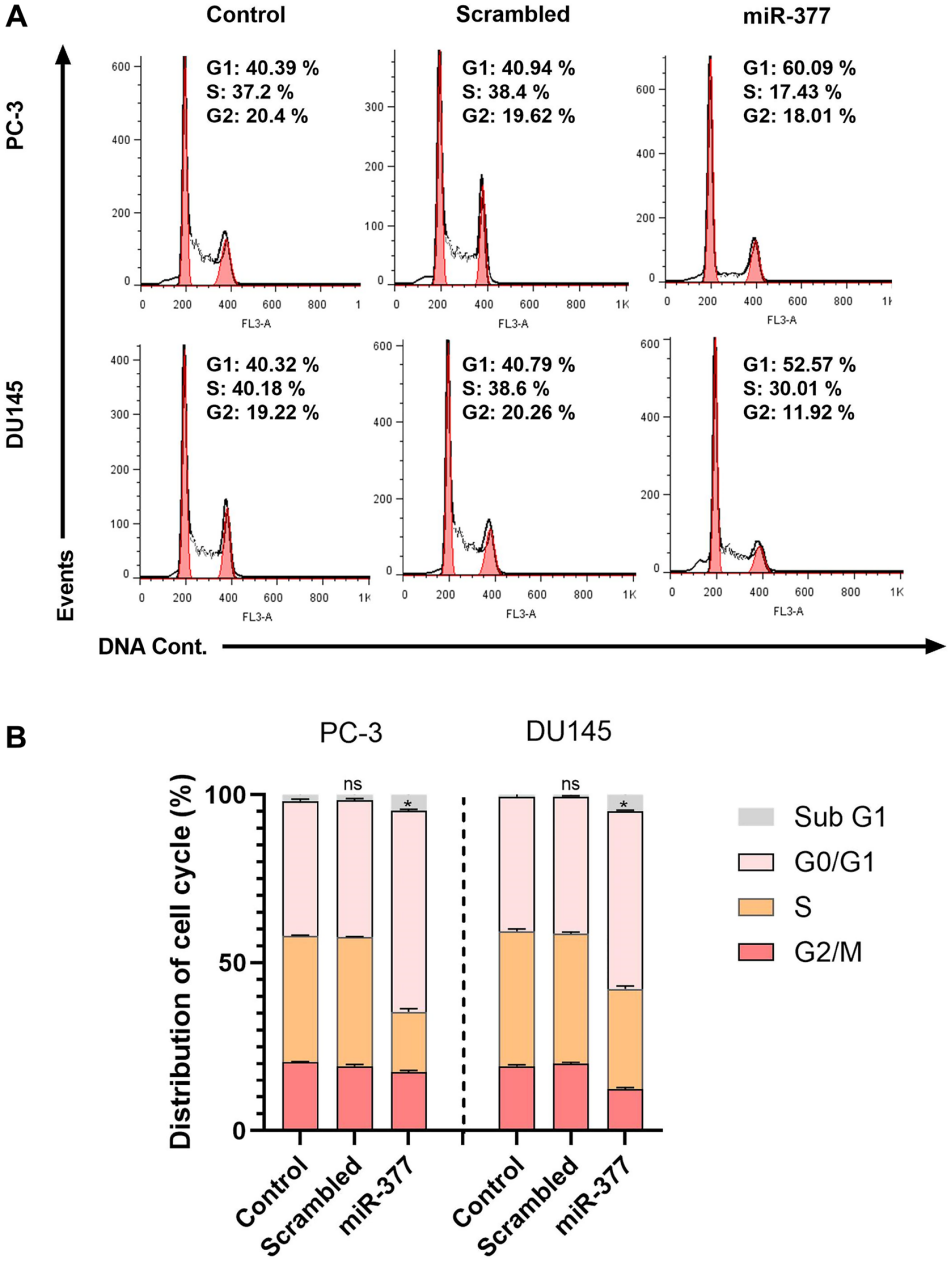
The effects of the miR-377 on the cell cycle of prostate cancer cell lines at the G0/G1 phase. (**A**) PC-3 and DU145 cells were transfected with miR-377 and subjected to PI staining before flow cytometry. Cell cycle stop was investigated using flow cytometry. (**B**) The graphs show that miR-377 could stop the cell cycle at the G0/G1 phase of PCa cell lines compared to miR-scrambled and control, ^*^P ≤ 0.05, Mean ± SD.

To examine the miR-377 function on the cell cycle, we transfected miR-377 in PCa cell lines. According to the FCM analysis of the cell cycle, overexpression of miR-377 prevented cells from progressing through the G0/G1 phase, halting the proliferation of cancer cells (Figure 4A, 4B).

### Inhibited cell cycle progression is caused by CDK4 down-expression

The TGCA samples showed that cancer tissues have a high level of CDK4 mRNA compared to normal tissues (Figure 2E). Also, Real-time PCR analysis of prostate cancer cell lines transfected with miR-377 revealed that the expression level of CDK4 mRNA was lower than that of control cells (0.341 ± 0.0225 in PC-3 cells and 0.2915 ± 0.024 in DU145). Based on prior studies and our real-time PCR analysis findings, it has been shown that miR-377-transfected PC-3 and DU145 cell lines exhibit decreased expression of CDK4 as a consequence of decreased MYC expression. When the cell is getting ready to begin DNA synthesis, CDK4 mediates progression through the G1 phase. Cyclins and the serine/threonine cyclin-dependent kinases, or CDKs they are connected with, play a role in part-regulating the cell cycle. When the cell is ready to synthesize DNA, CDK4 facilitates passage through the G1 phase with the D-type cyclins.

## DISCUSSION

With an annual incidence of over 160,000 cases, prostate cancer represents the most frequently diagnosed malignancy among males. Despite its generally slow-growing nature, prostate cancer remains the third-leading cause of cancer-related mortality in males [18]. Alterations of chromosome 8, particularly amplification at 8q24 involving the MYC oncogene, have emerged as one of the most common chromosomal abnormalities in the development of prostate cancer [19]. MYC as a proto-oncogene, is frequently overexpressed in prostate cancer, including the most advanced and metastatic castrate-resistant cases [20].

The dysregulation of miRNA, a group of small endogenous regulatory RNAs that range from 20 to 22 nucleotides in size, is a crucial factor in the development of tumors and is increasingly being acknowledged as a potential cancer biomarker for early detection and treatment strategies [21]. Previous investigations on lung cancer and pancreatic have demonstrated that miR-377 exhibits reduced expression in tumor samples when compared to controls [10, 11, 22]. Furthermore, it has been observed that miR-377 expression is significantly diminished in prostate cancer tissue. This decrease in expression is associated with the proliferation, apoptosis, migration, and invasion of metastatic prostate cancer cells [23].

The current investigation is primarily focused on the impact of the downregulation of miR-377 in prostate cancer cells, as previously demonstrated in studies [23, 24]. Specifically, our study aims to elucidate the functional relationship between miR-377 and the 3′-UTR region of MYC in PC-3 and DU145 cells. By utilizing luciferase reporters, our data confirms that the 3′-UTR of MYC serves as a target region for miR-377 in both PC-3 and DU145 cells. Numerous studies have highlighted the association between miR-377 downregulation and various human cancers, including lung tumors [25], hepatocellular carcinoma [26], osteosarcoma [27], clear cell renal cell carcinoma [28], glioblastoma [29], malignant melanoma [30], pancreatic cancer [11], and ovarian cancer [31].

We also examined the levels of MYC mRNA expression in PC-3 and DU145 cells after miR-377 transfection to confirm that it could target this mRNA. Results indicated that miR-377 can decrease the MYC gene’s mRNA expression level.

Moreover, the MYC gene has been shown to interact with other genes, such as BCL-2/Bax, PTEN, and CDK4. The anti-apoptotic protein encoded by the BCL-2 gene inhibits cell cycle progression [32]. MYC can upregulate BCL-2 expression, allowing malignant cells to avoid apoptosis and survive. BCL-2 gene expression is activated via MYC-mediated transcriptional activation. MYC gene overexpression can sometimes sensitize cancer cells to BCL-2 inhibitor-induced apoptosis. This is caused by disrupting the equilibrium between pro- and anti-apoptotic proteins, which activates the apoptotic pathway [33].

The BCL-2 protein, which is responsible for the regulation of apoptosis, functions by permeating the mitochondrial membrane and acting as a potent inhibitor of the apoptotic process [34]. The overexpression of BCL-2 has been observed in approximately 50% of all human malignancies. Furthermore, the interaction between BCL-2 and Bax, which is the pro-apoptotic component, results in the formation of a heterodimer. This contact between the two proteins leads to the elimination of Bax’s pro-apoptotic properties [35]. Therefore, the occurrence of apoptosis in a cell is contingent upon the proportion of BCL-2/Bax. It was observed that the introduction of miR-377 resulted in a notable decrease in the presence of BCL-2 mRNA in both PC-3 and DU145 prostate cancer cells. Conversely, exposure of these cells to miR-377 led to an elevation in the expression of the Bax protein. This outcome indicates that the Bax to BCL-2 ratio shifted towards apoptosis due to the decline in MYC mRNA levels.

The PTEN gene functions as a suppressor of tumors by exerting a negative regulatory effect on the PI3K/AKT signaling pathway, which is known to play a crucial role in cellular growth and survival [36, 37]. The regulation of cell growth, proliferation, and survival in cancers is associated with the molecular interactions between the MYC gene and PTEN.

Overexpression of the MYC gene can result in the downregulation or loss of PTEN expression, activating the PI3K/AKT pathway, thereby facilitating cell growth and survival [38]. In this research, we showed that the transfection of miR-377 in prostate cancer cell lines causes a decrease in MYC expression, and the deduction of the mRNA level of MYC causes an increase in the mRNA level of PTEN, and consequently, survival and proliferation of prostate cancer cell lines decrease.

Cyclin-dependent kinase 4 (CDK4) regulates the cell cycle and interacts with the MYC gene in this process. It participates in the transition from the G1 to the S phase of the cell cycle [39]. Overexpression of the MYC gene can result in the upregulation of CDK4 expression, thereby facilitating cell cycle progression and cellular division [40]. In this study, we show that miR-377 transfection causes a stop in the cell cycle of prostate cancer cell lines due to the decrease in the expression level of MYC mRNA and, thus, the reduction in the expression level of CDK4 mRNA.

**Figure 5:**
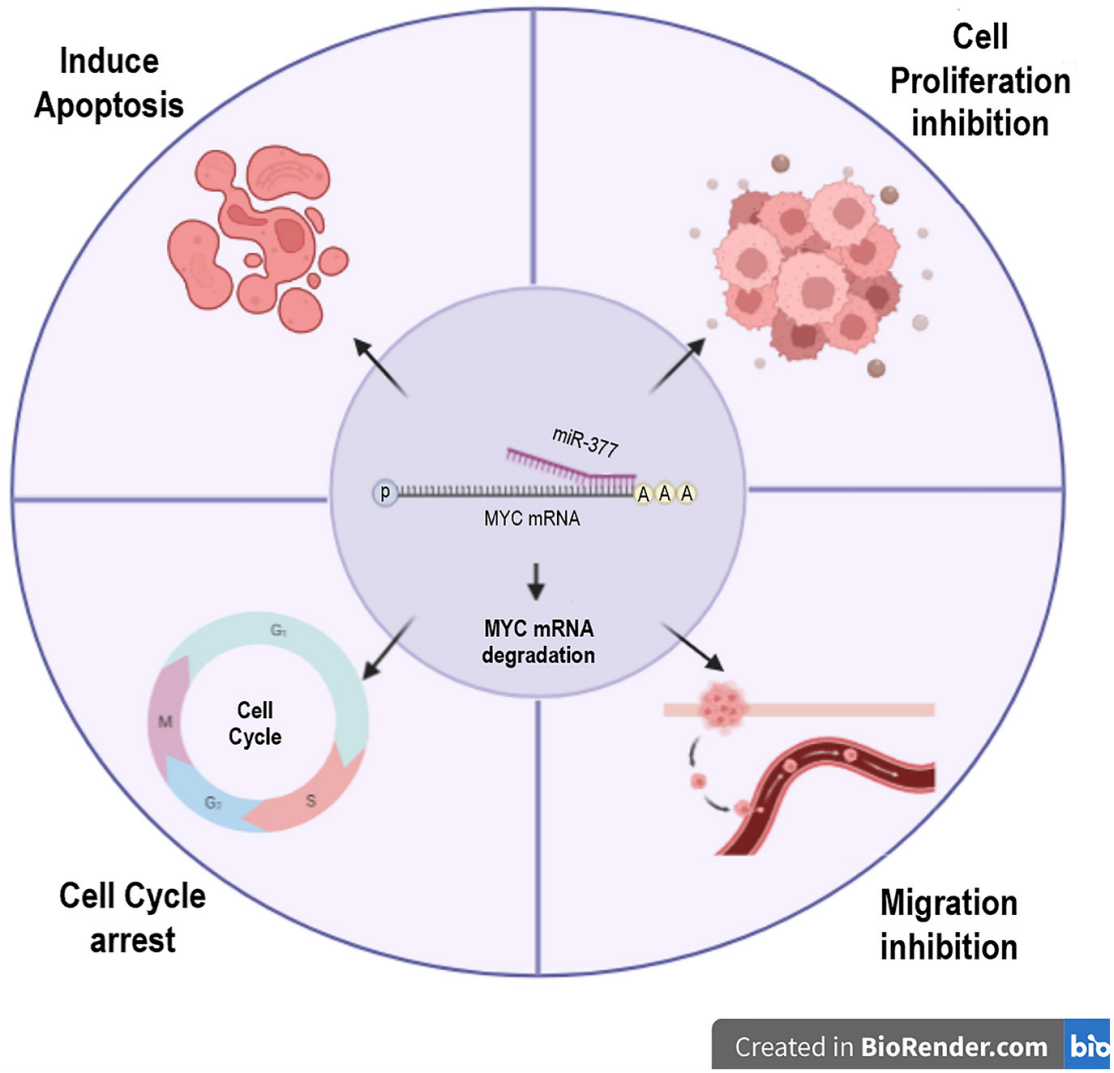
miR-377 transfection in prostate cancer cell lines can target MYC 3′UTR, degrade its mRNA, and affect apoptosis, cell cycle, migration, and cell proliferation.

The impact of miR-377 on cellular growth, apoptosis, and cell cycle was evaluated through the utilization of an MTT test and flow cytometry. The results demonstrated that miR-377 significantly impeded the cell proliferation, induced apoptosis, stopped the cell cycle at G0/G1 phase, and inhibited cell migration in PC-3 and DU145 cells when compared to the control and scrambled miRNA, suggesting that miR-377 may have a function in preventing prostate cancer by targeting MYC 3′UTR (Figure 5). These findings were consistent with earlier research that showed miR-377 inhibits the proliferation of lung cancer [10, 41], hepatocellular carcinoma [42], human osteosarcoma [43], glioblastoma [44], and pancreatic cancer cells [9, 22].

According to all available information, miR-377 is essential for prostate and other cancer cells to stop proliferation, cell cycle, migration and undergo apoptosis. Questions remain about the matching molecules in the above mentioned processes and other miR-377 targets.

In conclusion, we discovered that miR-377 regulates MYC mRNA in prostate cancer cell lines by targeting its 3′-UTR. MiR-377 transfection inhibits cellular proliferation, migration, and cell cycle and promotes apoptosis in PC-3 and DU145 cells, and this is due to the interaction of MYC with BCL-2/Bax, PTEN, and CDK4. Future research may discover more miR-377 targets to enhance understanding of the miR-377 regulatory network that drives PCa development. Additionally, our findings suggest that the therapeutic target miR-377 for the treatment of prostate cancer may be promising.

## MATERIALS AND METHODS

### MiR selection

Bioinformatics research was done to accept that miR-377 could target the 3′UTR of the MYC gene by using the miRBase, TargetScan, MiRanda, and miRWalk tools.

### Cell culture

The human prostate cancer cell lines (PC-3 and DU145) were obtained from the Cell Bank of the Pasteur Institute of Iran, Tehran, Iran. PC-3 cells were cultured in Roswell Park Memorial Institute (RPMI) 1640 medium, while DU145 cells were cultured in Dulbecco’s Modified Eagle Medium (DMEM) medium, supplemented with 10% fetal bovine serum, and antibiotics (10.000 units/mL of penicillin and 10.000 μg/mL of streptomycin) (all from Gibco; Thermo Fisher Scientific, Inc., Waltham, MA, USA) and maintained in a cell incubator with 5% CO_2_ at 37°C.

### MicroRNA transfections

A 24-well plate was seeded by 0.8 × 10^6^ cells one day before transfection. Using Lipofectamine 2000 (Life Technology, 11668019), according to the manufacturer’s instructions, cells were transfected with 100 nM of pre-miR precursors of miR-377 or control pre-miR precursors (scrambled) (Qiagen, Hilden, Germany) when their confluence reached 80%. The transfection medium was changed to a new medium containing 10% FBS after 6 hours. Cells from transfected lines were collected 24, 48, and 72 hours after transfection.

### Luciferase reporter assay

The 3′-UTR of MYC (NM_002467.6) was amplified for luciferase reporter studies using the primer pairs CCGCTCGAGAACTTGAACAGCTACGGAAC (forward) and ATAAGAATGCGGCCGCAGTCAGAGTCAAAGAAAGTAAT (reverse) (Endonuclease restriction sites are indicated by bold sequences). With 100 ng of genomic DNA, ten pmol of each primer, two μM MgCl2, 200 μM dNTP, and 1.5 units of Pfu polymerase, PCR was carried out in a total volume of 25 μL under the following conditions: 95°C for 5 min; 35 cycles of 95°C for 20 s, 51.5°C for 40 s, 72°C for 2 min and 45 s; and final extension target gene MYC’s 3′-UTR and a unique scrambled sequence (AAGCTTCATAAGGCGCATAGC) were cloned into the psiCHECKTM-2 Vector (Promega, C8021) just downstream of Renilla luciferase’s stop codon. Dual-Glo Luciferase Assay System was used to conduct luciferase experiments. One day before transfection, 4–5 × 10^4^ PC-3, and DU145 cells were grown in each well of a 48-well plate to perform the test. Following the manufacturer’s instructions, the cells were transfected using Lipofectamine 2000 reagent (Life Technology, 11668019) with 400 ng of psiCHECKTM-2 Vector (Promega, C8021) just 3′-UTR or scrambled sequence, and 100 nM of each pre-miR precursors (377 or scrambled miRNAs). Renilla luciferase activity in the multi-well plate luminometer was standardized to firefly luciferase activity. A dual luciferase reporter assay kit measured luciferase activities 24, 48, and 72 hours after transfection (Promega, USA).

### RNA isolation, cDNA synthesis, and RT-qPCR

The miRNeasy Mini kit (Qiagen, Germany) extracted total RNA from PC-3 and DU145 after transfection with each pre-miR precursors (miR-377 and miR-scrambled). Total RNA’s quantity and quality of total RNA were determined by Utilizing a UV spectrophotometric (IMPLEN, Munich, Germany) measure. The cDNA of the mRNA was generated using the cDNA synthesis kit (Fermentas, MA, USA). GAPDH (Table 1) was used as the endogenous control during the real-time PCR process. The SYBR Green real-time PCR Master Mix (Life Technology, MA, USA) 7.5 μL, 1 μL of cDNA, 0.5 mL of Forward Primer (10 pmol), 0.5 μL of Reverse Primer (10 pmol), and 5.5 μL of RNase-free H_2_O were combined to create the PCR mixes in 15 μL volumes. The statistical analysis for relative mRNA expression was conducted using the Relative Expression Software Tool (REST), as proposed by Pfaffl.

**Table 1:** Relative primers used for real-time PCR

Gene name	Primer sequence	Product size
**MYC**	Forward: GTAGTCGAAAACCAGCCTCCC	116bp
	Reverse: TTCTCCTCCTCGTCGCAGTA	
**BCL-2**	Forward: TCGCCCTGTGGATGACTGA	134bp
	Reverse: AGAGACAGCCAGGAGAAATCA	
**Bax**	Forward: TCAGGATGCGTCCACCAAGAAG	103bp
	Reverse: TGTGTCCACGGCGGCAATCATC	
**PTEN**	Forward: GCGGAACTTGCAATCCTCAG	133bp
	Reverse: TCACCACACACAGGTAACGG	
**CDK4**	Forward: GGCCTGTGTCTATGGTCGG	144bp
	Reverse: AGATCAAGGGAGACCCTCACG	
**GAPDH**	Forward: GAAAGCCTGCCGGTGACTAA	152bp
	Reverse: GCGCCCAATACGACCAAATC	

### Wound healing assay

A 12-well plate containing 0.5 × 10^6^ PC-3 and DU145 cells was seeded, and the cells were left to form a confluent monolayer overnight. After scraping the monolayer with a 10 mL pipette tip to eliminate any floating cells, washed three times with PBS, and the monolayer was then transfected with each pre-miR precursors (miR-377 and miR-scrambled). The cells were imaged at random locations inside each well, then incubated for 24 hours at 37°C. The cells were then captured on camera at the chosen locations for 24 hours. The initial and ultimate wound zones were considered while analyzing the cell migration.

### Apoptosis assay

One day before transfection, PC-3 and DU145 cells (3 × 10^4^ per well) were seeded into a 12-well plate. Following this, oligonucleotides, including pre-377 and miR-scrambled, were transfected using Lipofectamine 2000 (Life Technology, 11668019), by the manufacturer’s instructions, at a final concentration of 100 nM. According to the supplier’s instructions Annexin V-FITC/PI Apoptosis Detection Kit (Solarbio, CA1020), cells were collected after 48 hours and stained twice with FITC-labeled Annexin V and propidium iodide for flow cytometry analysis. The data generated was subjected to analysis using flow cytometry in the FL1 and FL3 channels on the Partec Flow cytometer.

### Proliferation assay

The assessment of cell proliferation was conducted using the MTT (Sigma, St. Louis, MO, USA). In this study, PC-3 and DU145 cell lines were utilized, with a seeding density of 3 × 10^4^ cells per well in 96-well microplates. These cell lines were subjected to transfection with either pre-miR-377 or a negative control. The cells that were in the logarithmic growth phase were collected and subsequently distributed onto a 96-well plate. 72 hours after the initiation of cell seeding, a volume of 10 μl of MTT was introduced into each well, followed by an incubation period of 4 hours at 37ºC. Each well was supplemented with 150 μl of dimethyl sulfoxide (DMSO), and the optical density (OD) was measured at a wavelength of 540 nm, with a reference wavelength of 630 nm. The viability of a sample can be determined by calculating the percentage using the formula: viability = 100 × (absorbance of the treated sample)/(absorbance of the control). The experiments were conducted in triplicate, and the mean values were computed. The experiments were replicated on three separate occasions, and the mean values were computed.

### Cell cycle assay

PC-3 and DU145 cells were plated in 24-well plates at a density of 1 × 10^6^ cells/mL to examine the impact of the miR-377 on the PCa cell cycle. The cells were transfected with scramble oligonucleotide or pre-miR-377 using Lipofectamine 2000 by the manufacturer’s instructions (Life Technology, 11668019). The cells were exposed to cold 70% ethanol for 24 hours at –20°C. After washing, the cells were stained with propidium iodide (PI) solution and allowed to air dry for 30 minutes. The cell cycle was investigated using flow cytometry.

### Statistical analysis

The mean and standard error are used to express results (SE). Data from each test were entered into GraphPad Prism V.9 for one-way ANOVA statistical analysis. All real-time PCR data were examined using the REST 2009 software and standardized for mRNA against GAPDH. *P*-value < 0.05 was considered significant.

## Abbreviations

Bax: BCL-2 associated X
BCL-2: B-cell lymphoma 2
CDK4: Cyclin-dependent kinase 4
MYC: Proto-Oncogene
BHLH: Transcription Factor
PCa: Prostate cancer
PTEN: Phosphatase and Tensin homolog
miR: micro-RNA
