# Correction: Inhibitory effect of miR-377 on the proliferative and invasive behaviors of prostate cancer cells through the modulation of MYC mRNA via its interaction with BCL-2/Bax, PTEN, and CDK4

**DOI:** 10.18632/genesandcancer.240

**Published:** 2025-01-29

**Authors:** Yasamin Azimi, Sara Hajibabaei, Ghazal Azimi, Fatemeh Rahimi-Jamnani, Masoumeh Azizi

**Affiliations:** ^1^Department of Molecular Medicine, Biotechnology Research Center, Pasteur Institute of Iran, Tehran, Iran; ^2^Department of Nanotechnology, Tehran Medical Branch, Islamic Azad University, Tehran, Iran; ^3^Department of Mycobacteriology and Pulmonary Research, Pasteur Institute of Iran, Tehran, Iran

Original article: Genes&Cancer. 2024; 15:28–40. 28-40. https://www.genesandcancer.com/article/236/pdf/

PMCID: PMC11098572 PMID: 38756697;

DOI: 10.18632/genesandcancer.236

**This article has been corrected:** The authors noticed a striking similarity between the apoptosis data presented in Figure 3C and the flow cytometry plots and gating percentages published in the authors’ previous article [1]. Upon investigation, they found that an honest mistake occurred during the preparation of Figure 3C, 3D, where flow cytometry data from the earlier article was inadvertently used. The flow cytometry plots mistakenly included in the manuscript do not accurately reflect the experimental conditions described in the present study. The authors have prepared corrected flow cytometry data and figures, along with an updated analysis, and have updated the text describing these results. These corrections do not change the conclusions of the article. The authors deeply regret this oversight and sincerely apologize for any confusion it may have caused.

The corrected version of the text of the article chapter and Figure 3 are provided below.

## Overexpression of miR-377 induced apoptosis in prostate cancer cell lines due to MYC down-expression

The impact of miR-377 on apoptosis in PC-3 and DU145 cells was assessed using annexin V and PI in flow cytometry. Compared to controls, the findings showed a rise in the number of cells going through early apoptosis. As shown in (Figure 3C, 3D), control cells for this experiment included un-transfected cells and cells transfected with scrambled oligonucleotides. In cells transfected with miR-377 compared to controls, the apoptosis ratio increased considerably. The percentage of early apoptotic cells increased from 1.05% to 16.6% in PC-3 and from 1.66% to 14.5% in DU145 transfected with miR-377.

**Figure 3:**
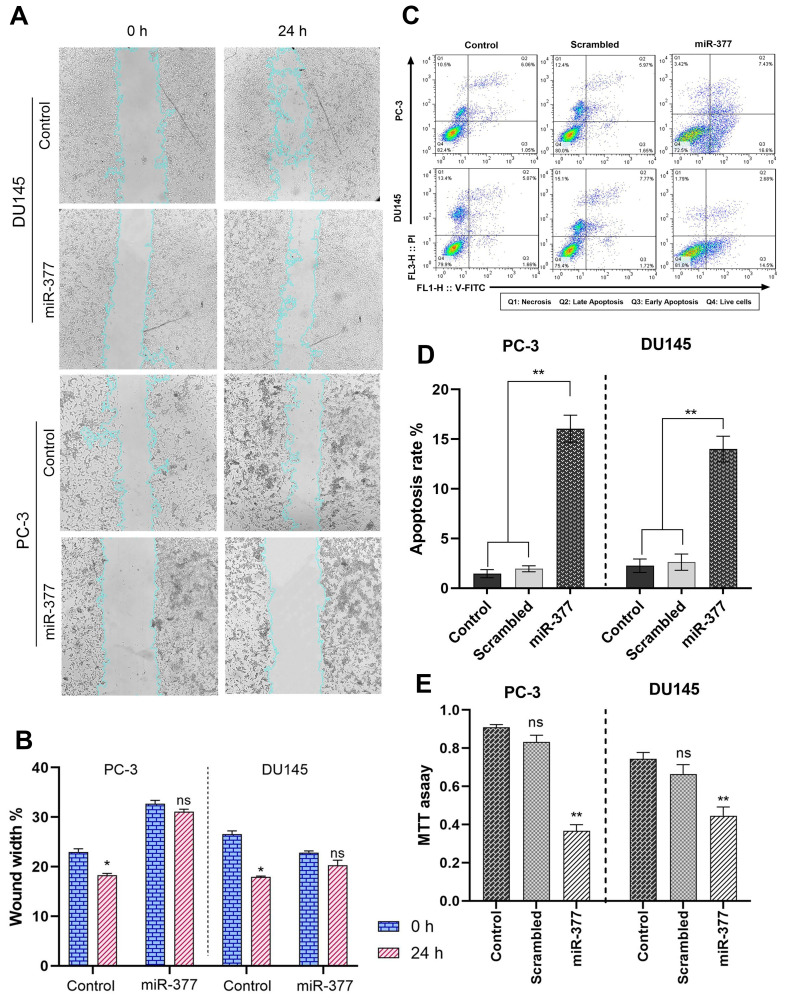
The effects of the miR-377 on cell migration, apoptosis, and MTT of prostate cancer cell lines. (**A**) Compared to control cells, representative images from wound healing assay of PC-3 and DU145 cell cultures 24 h after miR-377 transfection. (**B**) Wound healing assay graph of PC-3 and DU145 cell cultures 24 h after miR-377 transfection demonstrated that cell invasion into the cellfree region (outlined) is abatement compared to control cells. (**C**) Apoptosis induction was investigated using flow cytometry. (**D**) Apoptosis rates are shown in the graphs; Overexpression of miR-377 can induce apoptosis in PC-3 and DU145 cell lines. (**E**) The proliferation of PC-3 and DU145 cells transfected with miR-377, scrambled-miR oligonucleotide and control cells were determined using the MTT assay. Experiments were performed three times, and data are shown as mean ± SD. The results showed that miR-377 could markedly inhibit cancer cell proliferation of PCa cell lines, ^*^P ≤ 0.05, Mean ± SD.
